# The role of circulating anti-p53 antibodies in patients with advanced non-small cell lung cancer and their correlation to clinical parameters and survival

**DOI:** 10.1186/1471-2407-4-66

**Published:** 2004-09-14

**Authors:** Michael Bergqvist, Daniel Brattström, Anders Larsson, Patrik Hesselius, Ola Brodin, Gunnar Wagenius

**Affiliations:** 1 Department of Oncology, University Hospital, 751 85 Uppsala, Sweden; 2 Department of Medical Sciences, Clinical Chemistry, University Hospital, Uppsala, Sweden; 3 Department of Oncology, University Hospital of Huddinge, Stockholm, Sweden

## Abstract

**Background:**

Lung cancer causes approximately one million deaths each year worldwide and protein p53 has been shown to be involved in the intricate processes regulating response to radiation and/or chemotherapeutic treatment. Consequently, since antibodies against p53 (anti-p53 antibodies) are associated with mutations within the p53 gene it seems likely that these antibodies could, hypothetically, be correlated with prognosis.

**Methods:**

Serum samples from patients with non-small cell lung cancer (NSCLC) admitted to the Department of Oncology, University Hospital, Uppsala, Sweden, during 1983–1996 were studied. Anti-p53 abs were measured using a sandwich ELISA (Dianova, Hamburg, Germany).

**Results:**

The present study included 84 patients with stage IIIA-IV (advanced NSCLC). At least three serum samples from each patient were collected and altogether 529 serum samples were analysed for the presence of anti-p53 antibodies. The median value of anti-p53 antibodies was 0.06 (range 0 – 139.8). Seventeen percent of investigated NSCLC first serum samples (n = 84) expressed elevated levels of anti-p53 antibodies. Anti-p53 antibodies were not correlated to tumour volume or platelets.

Survival analysis showed that anti-p53 antibodies were not associated with survival as revealed by univariate analysis (p = 0.29). However, patients with adenocarcinoma had a significantly poorer survival if they expressed anti-p53 antibodies (p = 0.01), whereas this was not found for patients with squamous cell carcinoma (p = 0.13). In patients where the blood samples were collected during radiation therapy, a statistically significant correlation towards poorer survival was found (p = 0.05) when elevated anti-p53 antibodies levels were present. No correlations to survival were found for serum samples collected prior to radiation therapy, during chemotherapy, or during follow-up. When anti-p53 antibodies were measured continuously, no increase in median anti-p53 values was observed the closer the individual patient come to death.

**Conclusion:**

The result of the present retrospective study indicates that anti-p53 antibodies are not suitable for predictions concerning selection of patients with a more favourable outcome. Further prospective studies are, though, needed to fully elucidate this issue.

## Background

Lung cancer causes approximately one million deaths each year worldwide [1]. Treatment of these patients is based on surgery, but at diagnosis approximately 80 % of NSCLC patients are inoperable [2]. These inoperable patients are treated with radiotherapy and/or chemotherapy. Several studies have tried to improve survival through introducing new chemotherapeutic treatment combinations [3] or applying different radiation fractionation schedules [4], resulting in modest improvements in survival.

The continuous progress in the field of lung cancer biology has resulted in gradually increased insights into the intricate processes resulting in development and progression of malignancy. One of the first proteins whose occurrence was thought to affect prognosis was p53. This protein was identified during the late 1970s [5] and the corresponding gene was localized on the short arm of chromosome 17 [6]. Endogenous p53 protein is maintained at very low levels within the cell, but when the cell is exposed to hypothermia, oncogene activation, hypoxia or DNA damage, a rapid elevation of p53 levels is found, resulting in cell cycle arrest, DNA repair or induction of apoptosis [7]. If mutations are present in the p53 gene, these functions might be disturbed. In patients with NSCLC, p53 mutations are common, with mutation frequencies between 45–75% [8,9].

The question if mutations within the p53 gene are correlated to radiosensitivity is not defined. In an in vitro study from our group, we found that p53 mutations located in exon 7 were associated with significantly higher radiosensitivity than in those cell lines expressing p53 mutations in other exons [10]. In a clinical study performed on breast cancer patients with axillary lymph node metastases, the authors found that irradiation prolonged life in patients with p53 mutations compared with patients with wild type p53 [11].

Antibodies against p53 can be detected in sera from patients with cancer and a correlation exists between mutations in the p53 gene and antibodies against p53 in sera [12]. In a study from our group, investigating 67 patients with NSCLC, we found that the presence of anti-p53 antibodies prior to radiation therapy predicted increased survival (p = 0.025) [13].

In the present study, we included 84 patients with stage III-IV, donating 529 serum samples with the intention to investigate if predictions concerning outcome can be made and to investigate whether or not increased amounts of anti-p53 antibodies develop during disease progression, a question that, according to our knowledge, has not been explored previously.

## Methods

Serum samples from patients with NSCLC admitted to the Department of Oncology, University Hospital, Uppsala, Sweden, during 1983–1996 were studied. All patients gave informed consent prior to the collection of blood samples and the samples were stored at -70°C until analyzed. The study was reviewed and approved by the research ethics committee, Uppsala University, Uppsala, Sweden. The inclusion criteria were: a verified histology of NSCLC, advanced NSCLC defined as stage IIIA-IV according to TNM and a minimum of three serum samples donated from each patient during progression of the disease. The patients included were all followed from admittance until death.

The first serum samples from 67 patients included in this study had previously been investigated for the presence of antibodies against p53 [13]. In the present study, only patients with stages III and IV were included and the number of serum samples was enlarged with serum samples obtained during follow-up.

The clinical parameters investigated were: age, gender, histology, performance status, weight reduction, smoking, tumour volume (according to RECIST criteria), objective response, subjective response (defined from the clinical charts as either: feeling better after treatment or feeling worse after treatment) and the presence of anti-p53 antibodies. Complete information concerning clinical parameters could not be obtained due to inadequate information in the clinical charts.

### Anti-p53 antibody investigation

Blood was collected in 7 ml serum tubes without additive (367609, Becton Dickinson, Rutherford, NJ). Anti-p53 abs were measured using a sandwich ELISA (Dianova, Hamburg, Germany). Human recombinant p53 was bound to microtiter plates. Standards and samples were pipetted into the wells. After incubation and washing, a horseradish peroxidase conjugated polyclonal goat anti-human IgG was added. After renewed incubation and washing, a chromogenic substrate was added and the colour intensity was measured at 450 nm in a Titertek Multiskan. A relative index for patient sera was calculated as follows: E450 (sample) - E450 (low control)/E450 (high control) - E450 (low control). The ELISA assays were performed without knowledge of clinical data. According to the manufacturer's instructions, serum samples with an anti-p53 antibody index >1.1 were considered positive, whereas a serum sample with an index <0.9 was considered negative. Serum samples with an index between 0.9–1.1 were considered intermediately positive.

### Statistics

Survival was estimated using the Kaplan-Meier product limit method, where univariate analysis was performed using a log-rank test. Cox regression analysis was performed to investigate if certain continuous factors had a significant effect on survival or to perform multivariate survival analyses. Spearman's rank order correlation was utilised for tests of associations between factors. The survival analysis together with the descriptive statistics is based on the first serum sample collected for each patient, whereas the correlation analyses were performed using all sera samples.

In order to investigate if the levels of anti-p53 antibodies increased during the progression of the disease a statistical model was designed. In the model, time zero was set to be the date of pathological diagnosis and time one was set to time of death, i.e. the time to death was standardized for all patients. Using a fixed effect leased square estimator to allow for individually different starting values of anti-p53 antibodies, the effect on anti-p53 antibodies from diagnosis to death was calculated.

In the descriptive statistics, range was defined as the minimum and maximum. Throughout the paper a 5% significant level was used.

## Results

The median value of anti-p53 antibodies was 0.06 (range 0 – 139.8). Seventeen percent of the investigated NSCLC first serum samples expressed elevated levels of anti-p53 antibodies according to the manufacturer's instructions. From the time of pathological diagnosis until the time of death, no statistically significant effect on levels of anti-p53 antibodies was found (p = 0.8).

Anti-p53 antibodies were not correlated to tumour volume (p = 0.19) or platelets (p = 0.27). A numerically higher median value of anti-p53 antibodies was found for adenocarcinoma patients (index level: 0.12) than for squamous cell carcinoma patients (index level: 0.08).

**Figure 1:**
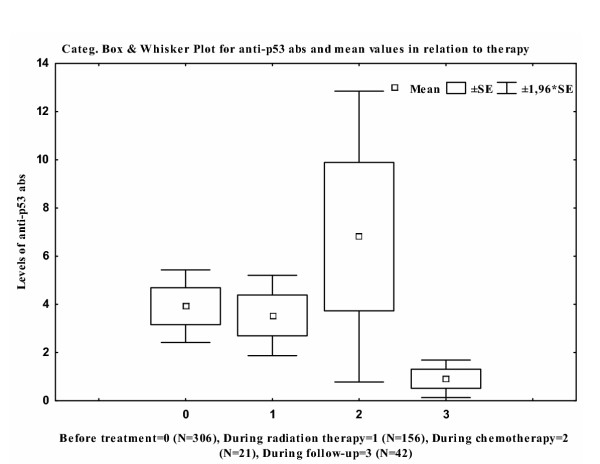
Distribution of anti-p53 antibodies according to treatment and the number of investigated serum samples are defined in the figure text

The median anti-p53 antibody level, prior to therapy, was statistically significantly elevated in comparison with serum samples were collected during follow-up (p < 0.001) (Fig 1). No statistically significant difference was found when the other groups were compared.

Descriptive data including survival, median and range for anti-p53 antibodies, as well as univariate analysis (based on the first serum sample for each patient), are shown in Table 1.

Analysis concerning histology and the presence of anti-p53 antibodies showed that patients with adenocarcinoma had a significantly poorer survival if they expressed anti-p53 antibodies (n = 23, p = 0.01). This was not found for patients with squamous cell carcinoma (n = 59, p = 0.13).

Survival analysis based on when the first serum sample was collected in relation to therapy revealed that anti-p53 antibodies collected prior to radiation therapy (continuous variable) were not associated with survival (n = 53, p = 0.14). In patients from whom blood samples were collected during radiation therapy, a statistically significant correlation towards poorer survival was found (n = 13, p = 0.05). However, no correlations to survival were found when the first serum sample was taken during chemotherapy (5 patients), or during follow-up (n = 12, p = 0.68).

**Figure 2:**
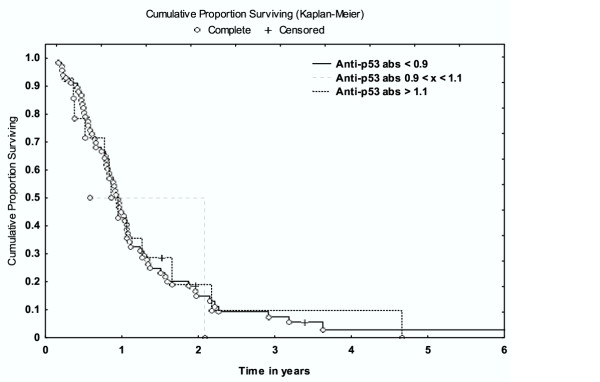
Survival analysis and the presence of anti-p53 antibodies

Survival analysis showed that increased amounts of anti-p53 antibodies were not associated with survival according to univariate analysis (p = 0.29) (Fig 2).

## Discussion

In the present study, anti-p53 antibodies have been investigated and correlated to various clinical parameters with the intention of studying if predictions concerning a more favourable outcome can be made for NSCLC patients, based on the presence or absence of these antibodies.

Correlation analysis showed that anti-p53 antibodies were not correlated with tumour volume (measured according to RECIST criteria). These results support results of a previous study from our group, in which we neither did find a correlation between anti-p53 antibodies and tumour volume in patients with NSCLC prior to thoracic surgery [14].

The clinical utility of anti-p53 antibodies in NSCLC patients with advanced disease has not yet been defined. In a previous study by our group, the presence of anti-p53 antibodies did not correlate to survival but the presence of anti-p53 antibodies prior to radiation therapy resulted in increased survival for anti-p53 positive patients [13]. In the present study, patients from the previous study were included and the number of patients, as well as the amount of investigated serum samples, were increased. According to our knowledge, the present study is currently one of the largest studies in patients with advanced NSCLC concerning the amounts of investigated anti-p53 antibodies in sera. We were unable to find a correlation between survival and anti-p53 antibodies (p = 0.29) but differences in survival were observed between the different histologies and the presence of anti-p53 antibodies. Patients with adenocarcinoma had poorer survival if expressing anti-p53 (p = 0.01), whereas this was not found for squamous cell carcinoma patients. In a study by Gao et al., examining p53 mutations through exons 5–8 in patients with squamous cell carcinoma and adenocarcinoma of the lung [15], it was shown that patients with adenocarcinoma expressed mutational hotspots at codons 248 and 249, whereas patients with squamous cell carcinoma had mutational events spread throughout exons 5–8. Further, the authors concluded that mutations in the p53 gene in adenocarcinoma patients resulted in production of a more rigid p53 protein. In the present study, although, no significant survival differences were found between these two histological subgroups, it may be speculated that immunogenic differences exist between adenocarcinoma and squamous cell carcinoma histologies, differences that might explain our results.

In the present study, the presence of anti-p53 antibodies was not correlated to survival when analyzed prior to radiation therapy, possibly because only patients with stages IIIA-IV were included, whereas our previous study also included patients with stages I-II. In patients where the first serum sample was collected during radiation therapy, a correlation to poorer survival was found (p = 0.05). Naturally, since the number of investigated patients was small, the obtained results should be interpreted with caution.

The issue of using anti-p53 antibodies in monitoring NSCLC patients has not yet been determined. Zalcman et al. used anti-p53 antibodies in NSCLC patients during follow-up in order to detect relapse [16], and concluded that anti-p53 antibodies in sera might be of clinical usefulness during follow-up of NSCLC patients. In the present study, serum samples were collected prior to, during, and after treatment. The distribution of serum samples was not homogeneously distributed during the different treatments, as seen in Fig 1. A change in antibody titer was observed during treatment and the levels of anti-p53 antibodies seemed to be higher during chemotherapy. These results should be interpreted with caution since the number of investigated serum samples was small. However, these results are intriguing, since anti-p53 antibodies have been correlated with the half-life of IgG1 and IgG2 [12]. Since patients receiving chemotherapy as well as radiation therapy often develop immunogenic deprivation, a subsequent reduction of anti-p53 antibodies seems reasonable, but this was not found. The results might be explained by tumour lysis causing increased exposure to the p53 antigen and, consequently, increased production of anti-p53 antibodies. Another hypothesis is that this elevation in the levels of anti-p53 antibodies during chemotherapy might be due to chemotherapy effects on normal tissues, thus explaining the difference between serum samples collected during radiation treatment and serum samples collected during chemotherapy treatment. A statistically significant difference was found between mean values of anti-p53 antibodies, prior to therapy and during follow-up (p < 0.001). However, since the investigated number of serum samples differs between the groups, no general conclusions can be made.

## Conclusion

The result of the present retrospective study indicates that anti-p53 antibodies are not suitable for predictions concerning selection of patients with a more favourable outcome. Further prospective studies are, though, needed to fully elucidate this issue.

## Authors' contributions

The authors have fulfilled the criteria for authorship according to rules stated and used in medical journals.

## Competing interests

None declared.

## Pre-publication history

The pre-publication history for this paper can be accessed here:



## Figures and Tables

**Table 1:** Descriptive data concerning survival (in days) as well as distribution of anti-p53 antibodies for investigated clinical parameters. The reported p-values relates to survival according to univariate analysis for the investigated clinical parameters

**Variables**	**Number of patients**	**Median Surv (days)**	SD*	**Anti p53 abs *Median/Range***	**P-value**
*Gender*					0.75
Male	62	330	582	0.08 (0–140)	
Female	22	363	257	0.1 (0–32)	
*Stage*					0.63
3a	47	324	660	0.02 (0–140)	
3b	25	332	203	0.23 (0–43)	
4	12	383	226	0.04 (0–25)	
*Performance status*					0.77
0	53	312	286	0.04 (0–43)	
1	21	359	392	0.23 (0–140)	
2	8	319	160	0.3 (0–32)	
*Smoking*					0.29
Yes	53	387	339	0.05 (0–140)	
Ex-smoker	22	294	228	0.24 (0–43)	
No	5	290	158	0.12 (0–6)	
*Weight reduction. kg*					0.78
Yes	42	348	293	0.20 (0–140)	
No	21	281	317	0.0 (0–10)	
*Histology*					0.73
Squamous carcinoma	59	324	321	0.08 (0–140)	
Adenocarcinoma	23	347	839	0.12 (0–25)	
Bronchioalv. ca	2	452	375	0.03 (0–0.05)	
*Tumour volume (recist)*					**0.007**
<50	18	446	433	0.04 (0–6)	
>50	21	290	142	0.22 (0–140)	
*Subjective improvement*					0.95
Yes	41	306	333	0.12 (0–140)	
No	11	306	146	0 (0–32)	
*Objective improvement*					0.73
Stable disease	5	551	371		0.06 (0–13)	
Regress of tumour	3	684	252	0 (0–0)	
Progress of tumour	2	526	333	0.55 (0–1.1)	
*Anti-p53 antibodies*					0.29
Continuous					
*Anti-p53 antibodies index*					0.96
neg	68	334	543		
intermediate	2	486	390		
pos	14	330	410		
